# A Handsome Biologic Handbook

**Published:** 1917-02

**Authors:** 


### A Handsome Biologic Handbook.

Every reader of this journal should write to the Abbott Laboratories, Chicago, for a copy of its beautiful new booklet, entitled "Biologic Remedies and How to Use Them." This is a good deal more than an advertising pamphlet; in fact, it is a real text-book of biologic therapy in which the essential facts are presented in simple, straightforward, untechnical English, making the topic intelligible and interesting to any physician. This booklet contains about 70 pages, and is illustrated with half-tone pictures and colored plates.

The first chapter presents the fundamental principles of biologic therapy; and there are also chapters describing the manufacture and use of bacterins, antitoxins, serums, and vaccines. If you want to know when to give a bacterin or antitoxin, how to inject it, the dosage proper in each case, the reaction likely to follow, or why you sometimes fail, you will get the help you want here. One of the most useful parts of the book is a department of Clinical Applications, in which the various disease conditions are taken up in alphabetical order and suggestions given for their treatment with biologic remedies. There is a very complete index, making the contents available for ready reference.

The book, as we have already said, will be sent free to anyone who will send his name to the Abbott Laboratories. Every user of biologic remedies should secure a copy and carry it in his pocket or satchel for help in emergencies.

